# Simple tuberculosis screening tool using signs, symptoms, and risk factors to reduce the missed opportunity in the older population

**DOI:** 10.1186/s12890-022-02001-2

**Published:** 2022-05-26

**Authors:** Agus Hidayat, Bhisma Murti, Soedarsono Soedarsono, Harsini, Chatarina Umbul Wahyuni, Isna Qodrijati

**Affiliations:** 1 Doctoral Program in Public Health, Graduate School, Universitas Sebelas Maret, Surakarta, Indonesia; 2 Department of Pulmonology and Respiratory Medicine, Dr. Soetomo General Hospital/Faculty of Medicine, Universitas Airlangga, Surabaya, Indonesia; 3 Master Program in Public Health, Graduate School, Universitas Sebelas Maret, Surakarta, Indonesia; 4 Department of Pulmonology and Respiratory Medicine, Faculty of Medicine, Universitas Airlangga, Surabaya, Indonesia; 5 Department of Pulmonology and Respiratory Medicine, Dr. Moewardi General Hospital/Faculty of Medicine, Universitas Sebelas Maret, Surakarta, Indonesia; 6 Department of Epidemiology, Faculty of Public Health, Universitas Airlangga, Surabaya, Indonesia; 7 Skills and Field Lab Unit, Faculty of Medicine, Universitas Sebelas Maret, Surakarta, Indonesia

**Keywords:** Tuberculosis, Sign, Symptom, Risk factor, Area under the ROC curve

## Abstract

**Background:**

There is a growing concern on how to increase tuberculosis (TB) case detection in resource-poor settings. The healthcare facilities routinely providing services to the elderly for chronic diseases often failed to detect TB cases, causing a missed opportunity. This study aimed to develop a simple and sensitive screening tool using signs, symptoms, and risk factors for TB case detection in the elderly.

**Methods:**

This was a cross-sectional study conducted from August to December 2020. A random sample of 302 subjects was taken from the elderly aged ≥ 60 years attending the outpatient polyclinic at Mangunharjo hospital, Madiun, East Java, Indonesia, for a chronic disease problem. The test was developed using 16 TB signs, symptoms, and risk factors. Test performance was assessed by comparing it against the GeneXpert MTB/RIF.

**Results:**

Marginal analysis resulted in the optimal cut-point of ≥ 7 for the test, which gave an area under the curve (AUC) of 0.62, with the maximum marginal AUC of 0.04 (*p* < 0.001). The sensitivity and specificity were 60.26% and 64.29%, meaning 60 TB cases for every 100 elderly with TB would be otherwise left undetected if this screening test did not take place.

**Conclusion:**

A simple TB screening tool with moderate sensitivity and AUC has been developed using TB signs, symptoms, and risk factors. It can be used as an initial step of the systematic TB screening in the elderly visiting healthcare facilities for routine chronic disease examination, with the additional utility of reducing the missed opportunity.

## Introduction

Tuberculosis (TB) has become one of the world's biggest public health concerns in the era of the aging population. The world’s TB burden is challenged by the shifting of TB from the younger to the elderly due to the aging population, longer life expectancy, and reactivation disease [1, 2]. Despite the decline in the overall global TB incidence, it remains an important problem among the older population in developed and developing countries. In the United States, persons 65 years of age and above shared the highest TB incidence in 2020 (3.4 per 100,000 persons) [3] In China, the prevalence of smear-positive tuberculosis in 2010 was 59 cases (49–72) per 100,000 population [2]. Globally, in 2010 most TB-related deaths occurred among persons aged 50 years or older with the majority in those aged 65 and above. In East Asia, older persons also share a large proportion of Disability-Adjusted Life Years (DALYs) with 51% of TB DALYs occurring in people aged 50 years and older [4]. In Indonesia, TB prevalence among the older population aged 65 and above in 2014 was as high as 1582 per 100,000 population [5]. In East Java province, Indonesia, the annual incidence of TB among people aged 65 and above in 2021 was 43,266 cases (8.1% of all age groups) [6].

The control of TB in the elderly remains a major challenge because of the limitations of the existing tools for the diagnosis and treatment of latent TB infection and clinically active disease [7]. There is a growing concern on how to increase TB case detection, especially among older adults in low and middle-income countries [4]. Sputum smear microscopy remains the mainstay of diagnosis. However, the difficulty to collect quality sputum and the atypical or lack of classical TB symptoms of in the elderly had led to TB cases being left undetected through the health care system. There are clear missed opportunities for earlier TB diagnosis, leading to delayed treatment initiation and continued spread of MTB to the community [8].

Several studies have developed a TB sign and symptoms screening tool (TB-SS screening test) using signs and symptoms such as cough, hemoptysis, loss of weight, chest pain, fever, night sweat, and shortness of breath. The TB-SS screening test serves as the first step for TB case finding with those who screen positive are recommended to have a chest X-ray (CXR) and acid-fast bacilli (AFB) sputum examinations [8]. Using signs and symptoms as an independent screening tool remains a global challenge for its relatively low accuracy. However, it could still be of value when it was used in a specific target population [10].

The current study aims to develop a simple TB screening tool that can be used in a resource-poor setting using a simple checklist questionnaire. The tool is different from the previous ones in that it uses a dual decision rule consisting of the selected individual TB signs, symptoms, and risk factors (TB-SSR) that demonstrate high sensitivity and aggregation of these TB-SSR with an optimal cut-point. The risk factors included TB contact history, lack of physical activity, smoking, alcohol drinking, co-morbidity, and previous TB treatment history. This screening tool is intended for use by clinicians as a complement when they are examining older patients for chronic disease problems at healthcare facilities. The complementary screening aimed to improve case detection, thereby reducing missed opportunities for TB case detection.

## Methods

This was an analytical study using a cross-sectional design. It was conducted at Mangunharjo hospital, Madiun, East Java, Indonesia, from August to December 2020 (5 months). The reference population was the older people aged ≥ 60 years attending the outpatient polyclinic at Mangunharjo hospital, Madiun, East Java, Indonesia, who did not receive any TB treatment. The dependent variable was the detection of TB cases using 16 independent variables, including TB signs, symptoms, and risk factors (TB-SSR). The sample size was estimated using the formula suggested by Hair et al. (2018) requiring a minimum of 15 study subjects per independent variable, resulting in a minimum sample size of 240 older persons (= 16 independent variables × 15 study subjects) [12]. Based on the number of previous out-patient visits to the elderly clinic, which was approximately 400 visits per month, a sampling frame was constructed consisting of 2000 patients (= 5 months × 400 patients). Google search engine was used to obtain 302 random numbers from the sampling frame of 2000 patients ranging from 0 to 1999. The order of patients in this sampling frame corresponded with the order of the actual patient visits. For example, a random number of 17 of the sample obtained means that the 17th patient visiting the elderly clinic was taken as a sample for this study. Likewise, a random number of 1700 means that the 1700th patient visiting the elderly clinic was taken as a sample.

Tuberculosis diagnosis was confirmed by sputum examination using the GeneXpert MTB/RIF. The GeneXpert MTB/RIF was chosen as the gold standard or reference for TB diagnosis as recommended by the World Health Organization [13]. GeneXpert MTB/RIF is a molecular test for the detection of TB and rifampicin resistance [14]. The sensitivity and specificity of GeneXpert MTB/RIF are high at 88% and 99%, respectively [13].

The screening test was developed using a combination of 16 different TB signs, symptoms, and risk factors (TB-SSR), which were pre-selected based on previous studies [7–9, 15]. TB-SSRs with corresponding operational definitions were as follows:

- Cough: persistent cough that lasts for 2 weeks or more with or without sputum.
- Hemoptysis: expectoration of blood alone or mixed with mucus.
- Dyspnea: intense tightening in the chest, difficult breathing, breathlessness, or a feeling of suffocation.
- Chest pain: abnormal pain in the chest when breathing or coughing.
- Chest tightness: heavy feeling in the chest.
- Night sweat: excessive perspiration at night.
- Loss of appetite: decreased desire to eat in the last 3 months.
- Weight loss: unintentional decrease in body weight in the last 3 months.
- Insomnia: persistent difficulty with sleep initiation, duration, or quality.
- Low activity: reduction of body movement ability to perform daily activities.
- Fatigue: physical or mental state of fatigue or lack of energy.
- Smoking: at least one stick cigarette smoking per day during the past 30 days.
- Alcohol consumption: habit of drinking alcohol containing beverage.
- Comorbidity: co-existing disease or medical condition (e.g. diabetes mellitus, asthma, chronic obstructive pulmonary disease, hypertension, arthritis, rheumatoid, cancer).
- TB contact: prolonged, frequent, or intense contact with TB infected person.
- History of TB treatment: previous medical TB treatment.

This study employed some performance indicators for the TB-SSR screening test, including sensitivity, specificity, accuracy, AUC (area under the Receiver Operating Characteristic curve), marginal AUC *p*-value, Odds Ratio, and *p*-value. An ideal test is expected to have a sensitivity and specificity of 80–90% [16]. Marginal AUC is defined as a change in the AUC as the cut-point increases one point.

The TB-SSR screening tool was developed alongside the following steps. Step 1: TB signs, symptoms, and risk factors (TB-SSR) were selected from previous studies. Step 2: A checklist questionnaire screening tool was constructed for collecting information on the TB-SSR. Step 3: GeneXpert MTB/RIF was chosen as the reference standard. Step 4: The clinicians interviewed each of all 302 patients to obtain information on whether or not they had any TB-SSR. Step 5: The clinicians filled in the response for each TB-SSR from the patients, and scored 1 if the TB-SSR was present and scored 0 if the TB-SSR was absent. Step 6: all 302 patients underwent GeneXpert examination. Step 7: Bivariate analyses were performed involving data of each TB-SSR and the results of the GeneXpert to calculate Odds Ratios, sensitivity, specificity, AUC, and p values. Since both TB-SSR and GeneXpert data were measured on a dichotomous scale, Chi-Square was used as the statistical test. Sixteen TB-SSRs showed statistical association with GeneXpert. Step 8: TB-SSRs that showed a sensitivity of ≥ 85% were adopted for use as criteria for the TB-SSR screening test classification scheme. The sensitivity rather than specificity was purposively used to decide whether or not a TB-SSR to be adopted into the screening tool since the screening was expected to minimize false-negative cases. Step 9: The 16 statistically significant TB-SSRs were added up to result in the total score of TB-SSR for each patient, with the total score ranging from 0 (minimum) to 16 (maximum). Step 10: To enable calculation of the test performance indicators, the continuous data of the total TB-SSR score were collapsed into a dichotomous scale using several cut-points ranging from 2 (lowest) to 15 (highest). Step 11: Using the cut-points of the total TB-SSR scores, the test performance indicators including sensitivity, specificity, accuracy, AUC, marginal AUC, marginal AUC *p*-value, Odds Ratio, and *p*-value were calculated. Step 12: The resulting statistics from Step 11 were arranged in a table from the lowest to the highest cut-points to enable marginal AUC calculation. Step 13: The marginal AUC was employed to determine the cut-point of the TB-SSR score that yielded the optimal AUC.

The present study employed a dual decision rule in selecting the criteria for the screening test classification scheme. The first decision rule was to select the TB-SSRs with a sensitivity of ≥ 85% as the screening test criteria [16]. The reason for choosing sensitivity rather than specificity is because the purpose of this effort is to develop a complementary screening test for use in a routine visit of older people at the healthcare facility for a chronic health problem, instead of a diagnostic test. A good screening test requires high sensitivity to minimize the possibility of false-negative cases.

The second decision rule in selecting the criteria for the screening test classification scheme was to use the optimal area under the curve (AUC) of the TB-SSR screening test. The AUC combines the information from sensitivity and specificity [17, 18]. The optimum AUC was determined by exploring AUCs with all possible cut-points. A cut-point that yields a larger AUC is preferred over a smaller one. The decision rule was to choose the cut-point at which the marginal AUC was maximized along with the lowest marginal AUC *p*-value. At this cut-point, the performance of the test is optimal as no further improvement can be made.

In addition to maintaining high sensitivity and improving the accuracy of the screening tool, the dual decision rule has the advantage to shun an alternative method of weight assignment for each TB-SSR, which otherwise would make the application of the screening tool cumbersome and time-consuming at the healthcare facilities.

Statistical analyses were performed using STATA version 13 (Stata Corp, College Station, TX, USA).

## Results

Table 1 shows that all of the sample members were the elderly with 86.4% of them aged 60–74 years, 64.2% male, 91.7% married, 62.6% retired/unemployed, and 90.7% living in their own house.

**Table 1:** Socio-demographic characteristics of the sample (n = 302)

Characteristics	Categories	n	%
Gender	Male	194	64.2
	Female	108	35.8
Marital status	Married	277	91.7
	Widow/widowed	25	8.3
Age (year)	60–74	261	86.4
	75–90	39	12.9
	> 90	2	0.7
Education status	Elementary school	47	15.6
	Junior high school	74	24.5
	Senior high school	141	46.7
	Bachelor	40	13.2
Employment	Unemployed/retired	189	62.6
	employed	113	37.4
Income	Rp 1,000,000 to < Rp 2,000,000	181	59.9
	≥ Rp 2,000,000	121	40.1
Residence	Own house	274	90.7
	Living in another house	28	9.3

Table 2 showed that 88.74% of the study subjects had a cough for ≥ 2 weeks, 3.97% were TB contact, 49.34% smoked, 42.72% had comorbidity, and only 4.64% had an alcohol drinking habit. The comorbidity included diabetes mellitus, chronic obstructive pulmonary disease, asthma, hypertension, atherosclerosis, cancer, dementia, and malnutrition. Sputum with good quality was sampled from all 302 patients for GeneXpert examination, 25.83% of whom were positive.

**Table 2:** Description of signs, symptoms, and risk factors (n = 302)

No	Signs, symptoms, and risk factors	Measurement	n	%
1	Cough ≥ 2 weeks	No	34	11.26
		Yes	268	88.74
2	Hemoptysis	No	274	90.73
		Yes	28	9.27
3	Dyspnea	No	119	39.40
		Yes	183	60.60
4	Chest pain	No	172	56.95
		Yes	130	43.05
5	Chest tightness	No	141	46.69
		Yes	161	53.31
6	Night sweat	No	176	58.28
		Yes	126	41.72
7	Loss of appetite	No	213	70.53
		Yes	89	29.47
8	Weight loss	No	138	45.70
		Yes	164	54.30
9	Insomnia	No	132	43.71
		Yes	170	56.29
10	Low activity	No	247	81.79
		Yes	55	18.21
11	Fatigue	No	126	41.72
		Yes	176	58.28
12	Smoking	No	153	50.66
		Yes	149	49.34
13	Alcohol consumption	No	288	95.36
		Yes	14	4.64
14	Comorbidity	No	173	57.28
		Yes	129	42.72
15	TB Contact	No	290	96.03
		Yes	12	3.97
16	History of TB treatment	No	261	86.42
		Yes	41	13.58
17	GeneXpert positive	No	224	74.17
		Yes	78	25.83

Table 3 shows that all TB-SSR had positive associations with the results of the GeneXpert MTB/RIF and all were statistically significant. The TB-SSRs that showed a sensitivity of ≥ 85% include cough (96.15%) and TB contact (89.7%), which according to the decision rule were taken as the TB-SSR screening tool classification criteria. Further, since all signs, symptoms, and risk factors were statistically significant, all of them were aggregated into the TB-SSR screening total score.

**Table 3:** The results of bivariate analysis relating each of the 16 signs and symptoms and the results of the GeneXpert MTB/RIF, showing the OR, 95% CI, and *p*-value, estimated from an elderly population (n = 302)

Independent variables	OR	95% CI		*p*	AUC	Sensitivity (%)	Specificity (%)
		Lower limit	Upper limit				
Cough	4.01	1.19	21.06	0.016	0.55	96.15	13.84
Hemoptysis	2.79	1.15	6.62	0.009	0.55	16.67	93.30
Dyspnea	1.79	1.01	3.28	0.037	0.57	70.51	42.86
Chest pain	1.68	1.01	2.82	0.049	0.56	52.56	60.27
Chest tightness	1.82	1.04	3.22	0.027	0.57	64.10	50.45
Night sweat	1.69	1.01	2.84	0.047	0.56	51.28	61.61
Loss of appetite	1.75	1.01	3.01	0.043	0.56	38.46	73.66
Weight loss	2.00	1.13	3.58	0.011	0.58	66.67	50.00
Insomnia	1.80	1.01	3.22	0.032	0.57	66.67	47.32
Low activity	1.86	1.01	3.47	0.048	0.55	25.64	84.38
Fatigue	1.74	1.01	2.99	0.046	0.57	67.95	45.09
Smoking	1.69	1.01	2.85	0.049	0.57	58.97	54.02
Alcohol consumption	4.15	1.21	14.96	0.006	0.54	10.26	97.32
Comorbidity	1.84	1.06	3.20	0.021	0.58	53.85	61.16
TB Contact	4.32	1.13	17.72	0.009	0.53	89.7	97.77
History of TB treatment	2.32	1.09	4.84	0.014	0.56	21.79	89.29

Table 4 describes the sensitivity, specificity, accuracy, AUC, marginal AUC, marginal AUC *p*-value, Diagnostic Odds Ratio, and *p*-value for all cut-points of the TB-SS screening test. These performance indicators are arranged in order from the lowest to the highest cut-points.

**Table 4:** Sensitivity, specificity, accuracy, AUC, marginal AUC, marginal AUC *p*-value, diagnostic odds ratio, and *p*-value for each cut-point of TB-SS screening test estimated from an elderly population as compared with the results of the GeneXpert MTB/RIF (n = 302)

Cut-point	Sensitivity (%)	Specificity (%)	Accuracy (%)	AUC	Marginal AUC	Marginal AUC *p*-value	Dx OR	*p*
≥ 2	98.72	3.13	27.81	0.51	–	–	2.48	0.398
≥ 3	94.87	8.48	30.79	0.52	0.01	0.570	1.71	0.341
≥ 4	83.33	22.77	38.41	0.53	0.01	0.526	1.47	0.258
≥ 5	73.08	36.61	46.03	0.55	0.02	0.389	1.57	0.122
≥ 6	61.54	54.02	55.96	0.58	0.03	0.186	1.88	0.019
≥ 7	60.26	64.29	63.25	0.62	0.04	< 0.001	2.73	< 0.001
≥ 8	52.56	73.21	67.88	0.63	0.01	0.730	3.03	< 0.001
≥ 9	48.72	80.80	72.52	0.65	0.02	0.184	4	< 0.001
≥ 10	41.03	88.39	76.16	0.65	0	0.977	5.3	< 0.001
≥ 11	29.49	93.30	76.82	0.61	− 0.04	0.091	5.83	< 0.001
≥ 12	14.10	97.77	76.16	0.56	− 0.05	0.012	7.19	< 0.001
≥ 13	7.69	99.55	75.83	0.54	− 0.02	0.114	18.58	0.007
≥ 14	3.85	100.00	75.17	0.52	− 0.02	0.129	1	–
≥ 15	1.28	100.00	74.50	0.51	− 0.01	0.155	1	–

The cut-point of ≥ 7 gives an AUC of 0.62 with the highest marginal AUC of 0.04 and the marginal AUC *p*-value of < 0.001. At this cut-point, the test's ability to discriminate between diseased and healthy subjects is optimal (no improvement can be made). At this cut-point, the sensitivity was 60.26% meaning that out of 100 TB subjects, 60 subjects were classified as positive by the test. That means, 60 TB cases for every 100 subjects with TB would be otherwise left undetected if this screening test did not take place for those visiting for a chronic disease problem, which would cause a missed opportunity. The specificity was 64.29% meaning that out of 100 TB healthy subjects, 64 subjects were classified as negative.

The OR was 2.73, meaning that the elderly visiting the clinic who experienced ≥ 7 signs and symptoms as detected by the SS-TB screening test were 2.73 times more likely to contract TB than those with signs and symptoms < 7, and this increased risk was statistically significant (*p* < 0.001).

**Fig. 1:**
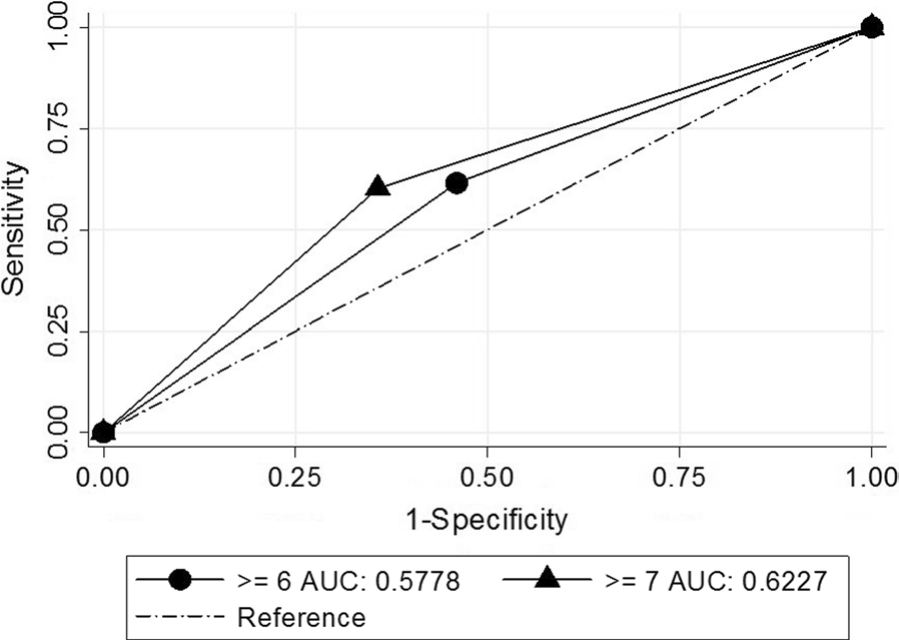
Receiver operating characteristic curve with AUC = 0.5778 for cut-point of 6 and AUC = 0.6227 for cut-point of 7

Figure 1 shows that the AUC = 0.6227 for a cut-point of 7 is larger than the AUC = 0.5778 for a cut-point of 6. Referring to Table 4, the marginal increase of one cut-point from a cut-point of 6–7 was 0.04, which is the highest of all marginal AUCs. This marginal AUC at cut-point 7 is statistically significant (*p* < 0.001). Thus, according to the decision rule, the optimal cut-point for the current SS-TB screening test is 7.

## Discussion

To the best of the authors’ knowledge, the present study is the first to develop simple TB signs, symptoms, and risk factors- based screening tool (TB-SSR screening test) using a dual decision rule involving sensitivity and AUC as the TB screening classification criteria. A marginal analysis approach was employed to determine the optimal cut-point for the TB-SSR screening test to classify positive and negative subjects. The optimal cut-point was chosen at which the marginal AUC was maximized. This discussion highlights several points.

Firstly, each TB-SSR in the current screening tool was significantly associated with the results of the GeneXpert MTB/RIF. This finding agreed with a study in Kenya, which used similar symptoms, including cough for > 7 days, productive cough, hemoptysis, fever for > 7 days, night sweat for > 7 days, and weight loss [19].

Secondly, an ideal screening test is expected to have a sensitivity and specificity of 80–90% [16]. However, conventional screening using any TB signs and symptoms generally suffered low sensitivity and specificity [9, 20, 21]. For example, the sensitivity of the four-symptom screening (including cough, weight loss, night sweats, or fever) for people living with HIV on antiretroviral therapy (ART) was as low as 51.0% (95% CI 28.4–73.2%) and the specificity was 70.7% (95% CI 47.8–86.4%) [20].

To address this issue, the present screening tool enhanced the sensitivity and specificity by incorporating not only TB signs and symptoms, but also risk factors including smoking, alcohol drinking, and TB contact (TB-SSR). Two TB-SSRs, i.e. cough ≥ 2 weeks and TB contact, were adopted for the screening criteria as they showed a sensitivity of 96.15% and 89.7%, respectively. For cough ≥ 2 weeks, it means 96 out of 100 subjects with GeneXpert positive cases would be otherwise left undetected if the test did not take place for those older persons visiting for a chronic disease problem, leading to a missed opportunity. Using either cough or TB contact alone provides much higher sensitivity than that of any combined TB signs and symptoms. For example, a study in Myanmar that used 32 various combinations of signs and symptoms resulted in a screening tool with a sensitivity of 59.8% (95% CI 54.1–65.3%) [9].

Thirdly, in addition to using the sensitivity of a TBB-SSR, the current TB-SSR screening tool also employed the AUC as the screening tool criteria. The AUC was calculated from the total score of 16 TB-SSRs, with the optimal cut-point selected at which the marginal AUC was maximized. The purpose of this combined criteria for the screening test is to improve the prediction power of the test by tapping additional information from the 16 TB-SSRs. With the cut-point ≥ 7, the AUC of the present TB-SSR screening tool was 0.62, which is comparable with those from the previous studies. For example, a TB screening trial in HIV-negative/unknown individuals conducted in South Africa reported an AUC of 0.68 (95% CI 0.64–0.72), whereas in the Zambian dataset the AUC was 0.66 (95% CI 0.60–0.72) [8]. A study in Zimbabwe that used symptoms including cough, hemoptysis, fever, night sweats, and weight loss, in the HIV-negative target population reported an AUC of 0.62 [15].

The strength of the current TB-SSR screening tool lies in the high sensitivity of cough ≥ 2 weeks and TB contact selected for the screening classification scheme. The limitation of this tool rests on the low to moderate AUC of the combined 16 TB-SSR, although it is comparable with some previous studies. The aggregated total score of the 16 TB-SSR also has skipped the weighting assignment to preserve the simplicity of the screening tool for use in real practice. Nevertheless, the importance of TB-SSR with high sensitivity (i.e. cough ≥ 2 weeks and TB contact history) have been individually accounted for in the screening tool.

## Conclusions

A simple and sensitive TB screening tool with low to moderate AUC has been developed using TB-SSR for TB case detection in the older population. By this screening tool, an individual with either cough ≥ 2 weeks OR TB contact OR the TB-SSR score ≥ 7 is classified as TB positive. Otherwise is TB negative. Those who are positive are recommended to have the confirmatory diagnostic processes.

This tool is not intended for use as an independent mass screening in a community-based active case finding. Rather, it is to be used as an initial step of the systematic screening for active TB in an older population who are visiting healthcare facilities for routine chronic disease examination. It has the additional utility of reducing the missed opportunity of detecting TB cases.

## Data Availability

The datasets generated and/or analyzed during the current study are not publicly available due to the privacy and confidentiality but are available from the corresponding author upon reasonable request.

## Abbreviations

AFB: Acid-fast bacilli
AUC: Area under the curve
CXR: Chest X-ray
DOR: Diagnostic odds ratio
HIV: Human immunodeficiency virus
LTBI: Latent tuberculosis infection
MTB: *Mycobacterium tuberculosis*
RIF: Rifampicin
ROC: Receiver operating characteristic
TB: Tuberculosis
TB-SS: Tuberculosis signs and symptoms
TB-SSR: Tuberculosis signs, symptoms, and risk factors
WHO: World Health Organization
